# Concordance between Patient Self-Reports and Claims Data on Clinical Diagnoses, Medication Use, and Health System Utilization in Taiwan

**DOI:** 10.1371/journal.pone.0112257

**Published:** 2014-12-02

**Authors:** Chi-Shin Wu, Mei-Shu Lai, Susan Shur-Fen Gau, Sheng-Chang Wang, Hui-Ju Tsai

**Affiliations:** 1 Department of Psychiatry, Far Eastern Memorial Hospital, New Taipei City, Taiwan; 2 College of Public Health, National Taiwan University, Taipei, Taiwan; 3 Department of Psychiatry, National Taiwan University Hospital and College of Medicine, National Taiwan University, Taipei, Taiwan; 4 Center of Neuropsychiatric Research, National Health Research Institutes, Zhunan, Taiwan; 5 Division of Biostatistics and Bioinformatics, Institute of Population Health Sciences, National Health Research Institutes, Zhunan, Taiwan; 6 Department of Medical Genetics, College of Medicine, Kaohsiung Medical University, Kaohsiung, Taiwan; 7 Department of Pediatrics, Feinberg School of Medicine, Northwestern University, Chicago, Illinois, United States of America; Technische Universitaet Muenchen, Germany

## Abstract

**Purpose:**

The aim of this study was to evaluate the concordance between claims records in the National Health Insurance Research Database and patient self-reports on clinical diagnoses, medication use, and health system utilization.

**Methods:**

In this study, we used the data of 15,574 participants collected from the 2005 Taiwan National Health Interview Survey. We assessed positive agreement, negative agreement, and Cohen's kappa statistics to examine the concordance between claims records and patient self-reports.

**Results:**

Kappa values were 0.43, 0.64, and 0.61 for clinical diagnoses, medication use, and health system utilization, respectively. Using a strict algorithm to identify the clinical diagnoses recorded in claims records could improve the negative agreement; however, the effect on positive agreement and kappa was diverse across various conditions.

**Conclusion:**

We found that the overall concordance between claims records in the National Health Insurance Research Database and patient self-reports in the Taiwan National Health Interview Survey was moderate for clinical diagnosis and substantial for both medication use and health system utilization.

## Introduction

The use of automated claims databases is a widely-used method for obtaining information for use in epidemiological studies. The advantages of claims-based studies compared with collecting information from patient reports or medical chart reviews include the inexpensiveness, large sample size, and lower risk of non-response [1], [2]. Taiwan's National Health Insurance Research Database (NHIRD), derived from the single-payer, compulsory National Health Insurance (NHI) program in Taiwan [3], is one of the largest available claims databases. As of 2007, about 22.6 million (98%) Taiwanese were enrolled. The NHI program covers ambulatory care, hospitalization and dental services, as well as preventive services. Patients' demographic characteristics, diagnoses, prescriptions, hospitalizations, and medical expenditures are recorded in the NHIRD. The number of published scientific papers using the NHIRD has increased dramatically in recent years [4]. However, since claims records are mainly generated to capture information for reimbursement purposes, physicians might record a diagnosis to receive payment when it is actually only just being considered. In contrast, a diagnosis which is not related to reimbursement might be under-recorded.

Patient self-report is a common method that is used to gather health information and that usually can provide other important data, such as the patient's perspective regarding their own general health conditions, lifestyle, and health behaviors. However, self-report has been shown to be affected by measurement error as the result of recall bias and the social desirability effect [5].

Both measurement methods have strengths and limitations. A linkage between claims data and patient self-reports could complement each other and enhance the utilization of both data sources in epidemiology and health services research. Despite the potential advantages, the concordance between claims data and patient self-report is still under-investigated in Taiwan. To date, only one study has examined the agreement between self-report and claims data on health system utilization, and the findings showed a fairly good concordance in the general population [6]. However, the concordance of clinical diagnoses and medication use in Taiwan remains unclear.

In this study, we used self-reported data collected from a nationwide representative survey, the 2005 Taiwan National Health Interview Survey (NHIS), to evaluate the concordance of clinical diagnoses, medication use and health system utilization, separately, between the self-reported data in the NHIS and claims records in the NHIRD. In addition, factors associated with disconcordance between the self-reported data and the claims records were also explored.

## Materials and Methods

### Study population

This study utilized data from the 2005 Taiwan National Health Interview Survey (NHIS), which is a nationwide cross-sectional survey used to investigate the health status of non-institutionalized residents in Taiwan. Detailed information related to the study design of the 2005 Taiwan NHIS can be found elsewhere [7]. In brief, households were randomly selected using a multistage stratified systematic sampling scheme. All members of these households were interviewed by trained interviewers using structured questionnaires. There were three versions of the questionnaires, one for each of the three age groups (<12, 12–64, and ≧65 years, separately). The questionnaire contained several domains, including sociodemographic characteristics, personal health status, health system utilization, and occupation and economic status. Most participants in the survey were interviewed from April to July 2005. A total of 30,680 residents was selected and 24,726 (80.6%) agreed to participate in the 2005 Taiwan NHIS. In this study, we included only those aged 12 years and above, yielding 20,826 participants. Of this group, 15,574 (75%) participants gave informed consent to allow us to link their NHIS data with the data of Taiwan's NHIRD for research purposes. After linkage, any information that could be used to identify the participants was anonymized and de-identified prior to analysis. The study was approved by the ethics committee of the National Health Research Institutes.

### Self-reported measures

We used information from the NHIS to measure self-reports in three domains, including clinical diagnoses, medication use, and health system utilization. Fourteen common clinical diagnoses, including hypertension, diabetes mellitus, dyslipidemia, malignancy, stroke, asthma, chronic pulmonary diseases, gout, osteoporosis, arthritis, renal disease, heart diseases, chronic hepatitis, and psychiatric disorders were explored in the versions of the questionnaires for the 12–64 and ≧65 years age groups. The participants were asked two sequential questions, “Do you have this disease?” and “Were you told by a doctor or a nurse that you have this disease?” For some diseases with an episodic course, such as asthma, the participants were asked a third question, “Did you have this disease during the last year?” If the answers to these questions were all ‘Yes', the patient was classified as having this disease. If the patient had any negative or uncontained answers for a question, we classified the patient as not having this disease.

For patients with any of the above-mentioned diseases, self-reported medication use was explored by asking, “Have you received any medication for treatment in the last year?” Given that some diseases were treated without specific medications, we analyzed the concordance in medication use by focusing only on five types of drugs, including antihypertensive drugs, antidiabetic drugs, lipid-lowering agents, anti-asthmatic drugs, and anti-gout drugs.

With regard to health system utilization, we asked, “Were you hospitalized during the last year?”; “Did you visit the emergency department during the last year?”; “Did you utilize dental services during the last year?”; and “Did you have a health examination though the NHI program during the last year?”

### Claims-recorded measures

The clinical diagnoses in the claims records were coded using the clinicians' judgment based on clinical history, diagnostic criteria, image, or biochemical data. The present analysis used NHIRD claims records for the one year preceding the 2005 Taiwan NHIS. We set May 15, 2005, which was approximately the median date of the survey, as the date for interviewing all participants. Both ambulatory and inpatient claims records for the period from May 16, 2004 to May 15, 2005 were extracted from the NHIRD for subsequent analysis. We used ICD9-CM codes in any diagnostic position, rather than only the primary position, to identify 14 clinical diagnoses from the ambulatory and inpatient claims in the NHIRD. Claims records from all types of healthcare settings were included. Among outpatient claims records during the study period, approximately 82.4% were Western Medicine, 7.8% were dental services, and 9.8% were Chinese Medicine. The target medications were identified using the Anatomical Therapeutic Chemical (ATC) classification system [8]. Given that a medication can be used for different indications, we defined a participant as a medication user only if the patient had a prescription record for a targeted medication with a corresponding diagnosis. For example, diuretics can be prescribed for hypertension or renal diseases; therefore, only those participants who received a prescribed diuretic and had a diagnosis of hypertension were defined as antihypertensive users. Regarding health system utilization, we assessed the type of medical services provided for each claims record in the NHIRD. The detailed ICD9-CM codes for clinical diagnoses and ATC codes for medication are provided in Table S1.

### Statistical analysis

The percentages for each item, clinical diagnoses, medication use, and health system utilization, based on self-reports or claims records were calculated by dividing the number of participants who had the presence of each item, separately, by the total number of participants. In terms of the overall percentage of each domain, the percentage was calculated as the average value for each item within the same domain. For the concordance between self-reports and claims records, we calculated the positive, negative, and total agreement and Cohen's kappa statistics, a chance-adjusted agreement [9], [10]. The interpretation for kappa was based on Landis and Koch's classifications: <0.2 as slight, 0.21–0.40 as fair, 0.41–0.60 as moderate, 0.61–0.80 as substantial, and 0.81–1.00 as almost perfect [11].

Given that physicians might initially code a tentative diagnosis and then change it later if further evidence did not support this initial diagnosis, the use of a strict algorithm, via increasing the number of claims records used to identify clinical diagnoses, is a common strategy to improve accuracy [8]. Therefore, we explored the concordance between self-reports and claims records using three algorithms to define the clinical diagnoses: ≧1 outpatient or ≧1 discharge claims, ≧2 outpatient or ≧1 discharge claims, and ≧3 outpatient or ≧1 discharge claims. The interval between two claims records was not specified; however, if two or more outpatient records were found on the same day, only one claims record was calculated.

Furthermore, we explored the factors of disconcordance between self-reports and claims records for diseases, medication use, and health system utilization. For each comparison, the outcome was dichotomized into agreement and disagreement between self-report and claims record. Explanatory variables included grossly trisected age groups (<30, 30–49, and ≧50), sex, educational level (≤6, 7–12, and>12), marital status, and urbanization level of residence (urban, suburban, and rural). Since the analyses were based on patient-condition pairs, we estimated the odds ratios and 95% confidence intervals using the multivariate generalized estimating equation with an unstructured covariance matrix to eliminate the effect of multiple representations of each patient. In addition to the above-mentioned general factors, we also explored the effect of disease-specific factors on certain clinical diagnoses. For example, obesity is strongly associated with hypertension, diabetes, and dyslipidemia. As such, we extract information on body mass index (BMI) from Taiwan's NHIS and categorized participants into normal (BMI<24), overweight (24≤BMI<27), and obese (BMI≧27). Using a logistic regression model, the effect of overweight and obesity on the agreement between hypertension, diabetes, and dyslipidemia were investigated.

SAS version 9.2 was used for all statistical analyses (SAS Institute, Cary, NC).

## Results

A total of 15,574 participants aged 12 years and above who consented to link their NHIS data with the NHIRD were included in the analysis. The mean age of participants was 41.2±18.7 years, 47.8% were females, 57.6% were married, 76.9% had more than 6 years of education, and 44.6% lived in urban areas. Compared with those who did not consent to link their NHIS data with the NHIRD, the consenters tended to be younger, more likely to be males, more highly educated, and more likely living in an urban area. However, the difference between consenters and nonconsenters was small (Table 1).

**Table 1:** Characteristics of study subjects with and without consent to data linkage.†

	Total sample	Consent	No consent
	(N = 20,826)	(N = 15,574)	(N = 5,252)
Age, years			
12–29	6,910 (33.2)	5,424 (34.8)	1,486 (28.3)
30–49	7,439 (35.7)	5,672 (36.4)	1,767 (33.6)
≥50	6,477 (31.1)	4,478 (28.8)	1,999 (38.1)
Gender			
Female	10,191 (48.9)	7,442 (47.8)	2,749 (52.3)
Male	10,635 (51.1)	8,132 (52.2)	2,503 (47.7)
Education, years ‡			
≤6	5,291 (25.4)	3,600 (23.1)	1,691 (32.2)
7–12	9,969 (47.9)	7,623 (48.9)	2,346 (44.7)
≥13	5,550 (26.6)	4,344 (27.9)	1,206 (23.0)
Marital status ‡			
Single	7,364 (35.4)	5,701 (36.6)	1,663 (31.7)
Married/living as married	12,263 (58.9)	8,977 (57.6)	3,286 (62.6)
Divorced/widowed/separated	1,156 (5.6)	862 (5.5)	294 (5.6)
Residential area			
Urban	9,313 (44.7)	6,944 (44.6)	2,369 (45.1)
Suburban	8,410 (40.4)	6,204 (39.8)	2,206 (42.0)
Rural	3,103 (14.9)	2,426 (15.6)	677 (12.9)

Note:

† All *p*-values for the chi-square tests between patients with or without consent were <0.001.

‡ A total of 16 participants had missing data for educational level and 43 participants had missing data for marital status.

The prevalence of most clinical diagnoses, medication use, and health system utilization based on claims records was higher than that based on self-report measures, except for dyslipidemia, osteoporosis, use of lipid lowering agents, and utilization of dental services (Table 2). The kappa varied across conditions, ranging from 0.18 for chronic pulmonary diseases to 0.85 for anti-diabetes drug use. The positive agreement ranged from 0.20 for chronic pulmonary diseases to 0.85 for anti-diabetes drug use. The total and negative agreement was high (all >0.8).

**Table 2:** Concordance between self-reports and claims records, by disease diagnoses, medication use, and health system utilization.

	Self-reports (%)	Claims records (%)	In claims records, in self-reports (%)	In self-reports only (%)	In claims records only (%)	Not in claims records, not in self-reports (%)	Total agreement	Positive agreement	Negative agreement	Kappa
**Diagnoses**										
Hypertension	11.6	12.7	8.8	2.7	3.8	84.6	0.93	0.73	0.96	0.69
Diabetes	4.7	5.7	4.0	0.7	1.7	93.6	0.98	0.77	0.99	0.76
Dyslipidemia	11.6	6.9	3.5	8.0	3.3	85.1	0.89	0.38	0.94	0.32
Malignancy	0.8	1.6	0.7	0.1	0.9	98.3	0.99	0.57	0.99	0.57
Stroke	1.2	2.5	0.8	0.4	1.7	97.1	0.98	0.43	0.99	0.43
Asthma	2.1	2.8	0.9	1.1	1.9	96.0	0.97	0.38	0.98	0.37
Chronic pulmonary diseases	2.4	5.3	0.8	1.6	4.5	93.1	0.94	0.20	0.97	0.18
Gout	3.5	4.2	1.9	1.6	2.3	94.2	0.96	0.50	0.98	0.48
Osteoporosis	3.7	1.9	0.8	2.9	1.1	95.2	0.96	0.29	0.98	0.27
Arthritis	3.3	7.0	1.6	1.7	5.4	91.2	0.93	0.31	0.96	0.28
Renal diseases	3.6	4.1	1.3	2.3	2.7	93.6	0.95	0.34	0.97	0.32
Heart diseases	3.9	8.2	2.6	1.3	5.6	90.5	0.93	0.43	0.96	0.40
Chronic hepatitis	3.2	7.1	2.0	1.2	5.1	91.7	0.94	0.39	0.97	0.36
Psychiatric disorders	1.6	6.8	1.0	0.6	5.8	92.6	0.94	0.23	0.97	0.21
Overall	4.1	5.5	2.2	1.9	3.3	92.6	0.95	0.46	0.97	0.43
**Medication use**										
Anti-hypertensives	9.7	11.3	7.8	1.9	3.4	86.9	0.95	0.75	0.97	0.72
Anti-diabetes	4.1	4.4	3.6	0.5	0.8	95.1	0.99	0.85	0.99	0.85
Lipid lowering agents	3.7	3.2	1.6	2.1	1.6	94.7	0.96	0.46	0.98	0.44
Anti-asthmatics	1.4	2.4	0.8	0.7	1.7	96.9	0.98	0.39	0.99	0.38
Anti-gout drugs	2.9	3.0	1.4	1.6	1.7	95.4	0.97	0.45	0.98	0.44
Overall	4.4	4.9	3.0	1.3	1.8	93.8	0.97	0.66	0.98	0.64
**Health system utilization**										
Hospitalization	7.0	8.0	5.2	1.8	2.8	90.2	0.95	0.69	0.98	0.67
Emergence room visit	13.4	16.4	9.1	4.3	7.3	79.3	0.88	0.61	0.93	0.54
Dentistry services	38.5	37.2	28.3	10.2	8.9	52.6	0.81	0.75	0.85	0.59
Health examination	6.7	8.0	3.2	3.5	4.8	88.5	0.92	0.44	0.96	0.39
Overall	16.4	17.4	11.4	4.9	6.0	77.7	0.89	0.68	0.93	0.61

**Figure 1:**
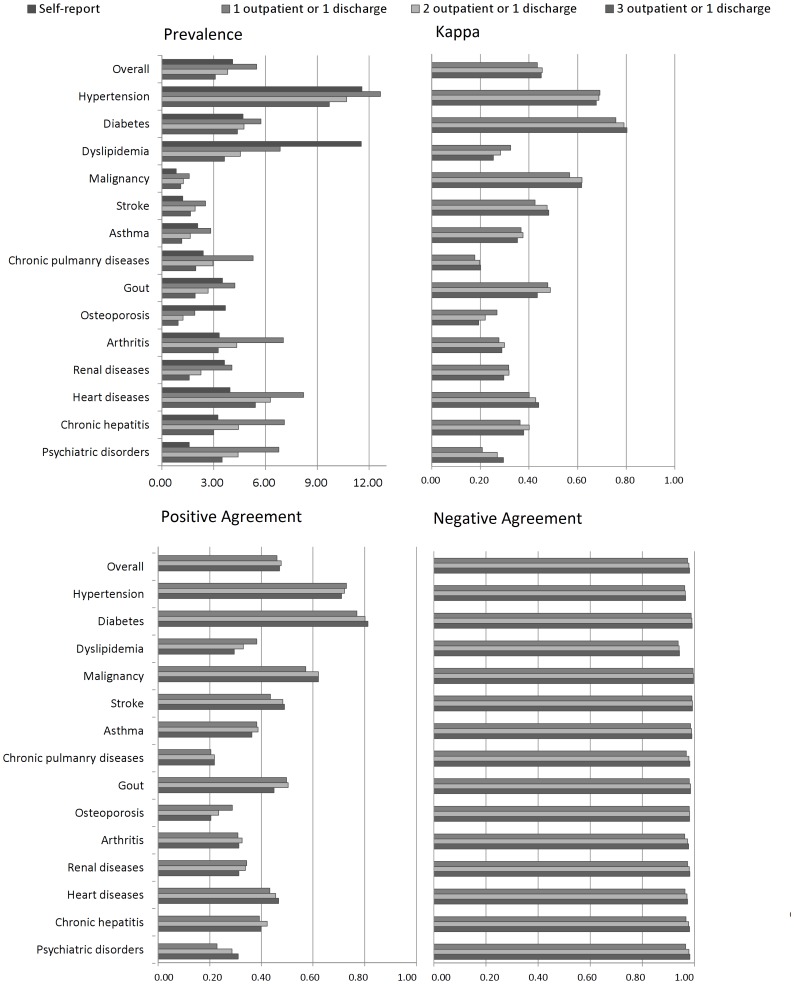
Comparison of the concordance between claims records and self-reports using different algorithms.

Using a strict algorithm for identifying clinical diagnoses in the claims records would have decreased the estimated prevalence. However, the effect on positive agreement and kappa was diverse across various conditions (Figure. 1).

In terms of factors associated with disagreement between self-reports and claims records, we found that old age was associated with more disagreement in clinical diagnosis, medication use, and health system utilization. Subgroup analyses for the three different age groups are shown in Tables S2–S4. Briefly, the prevalence, as well as the kappa, of most items increased with age; however, the total agreement decreased with age. Male gender was associated with disconcordance in medication use, but less disconcordance in health system utilization than in their female counterparts. There was less disagreement between self-reports and claims records of patients who were single or who had a high level of education. The level of urbanization of residency had little effect on disconcordance (Table 3). In terms of the effect of disease-specific factors on the concordance of clinical diagnosis, we found that obesity was associated with disagreement in hypertension, diabetes mellitus, and dyslipidemia but not in psychiatric disorders (Table S5).

**Table 3:** Factors associated with disconcordance between self-reports and claims records.

	Disease diagnosis		Medication use		Health system utilization	
	Odds ratio (95% CI)		Odds ratio (95% CI)		Odds ratio (95% CI)	
Age group (vs. 12–29)						
30–49	2.26	(2.02, 2.54)	3.17	(2.49, 4.04)	0.85	(0.77, 0.94)
≧50	5.75	(5.08, 6.51)	9.53	(7.38, 12.32)	1.21	(1.08, 1.36)
Gender						
Male vs. female	1.04	(1.03, 0.99)	1.51	(1.38, 1.65)	0.94	(0.89, 0.99)
Education (vs. ≤6 years)^a^						
7–12	0.71	(1.03, 0.66)	0.60	(0.53, 0.67)	0.82	(0.76, 0.89)
≧13	0.76	(1.04, 0.70)	0.49	(0.42, 0.57)	0.78	(0.72, 0.85)
Marital status^a^ (vs. single)						
Married/living as married	1.35	(1.06, 1.21)	1.15	(0.95, 1.41)	1.32	(1.19, 1.46)
Divorced/widowed/separated	1.25	(1.07, 1.08)	1.09	(0.84, 1.41)	1.18	(1.02, 1.37)
Residence (vs. urban)						
Sub-urban	0.97	(1.03, 0.91)	0.92	(0.84, 1.02)	1.03	(0.97, 1.09)
Rural	1.05	(1.04, 0.98)	1.01	(0.89, 1.14)	1.08	(1.00, 1.16)

## Discussion

In this study, using a nationwide representative survey to explore the concordance between self-reports and claims records for clinical diagnoses, medication use, and health system utilization, the results from kappa statistics showed that the overall concordance was moderate for clinical diagnosis and substantial for medication use and health system utilization. In addition, negative agreement, an analogue of specificity, was quite high for most conditions.

We found that the concordance of chronic diseases, such as hypertension and diabetes mellitus, was substantial. These two diseases had clear diagnostic criteria and are well-known in the general population. Thus, the concordance of these two diseases, as well as those of anti-hypertensive and anti-diabetes drug use, was quite high. However, the concordance of stroke, malignancy, and gout were only moderate. The concordance for serious diseases, such as malignancy or stroke, was lower than our expectation. There are several possible explanations for this. The low prevalence of these serious diseases might affect kappa statistics [9], and the lack of an exact timeframe in the interview questions might also be responsible for the observed low concordance. Patients might have had these serious diseases in the past and recovered from them in recent years; therefore, they may have not visited clinics in the past year because of these medical conditions. We also observed that the concordance of gout was only moderate. Gout causes sudden and severe pain; however, given the episodic nature of gout, patients might not recall exactly.

Furthermore, we found that the concordance of dyslipidemia, asthma, chronic pulmonary diseases, osteoporosis, arthritis, renal diseases, heart diseases, chronic hepatitis, and psychiatric disorders were only fair. In fact, we found that the concordance of dyslipidemia was lower than our expectation. The prevalence of dyslipidemia based on self-reports (11.9%) was similar to the finding for the blood lipid examination in the Nutrition and Health Survey in Taiwan, which showed that the prevalence of hypercholesterolemia was 10.2% in men and 11.2% in women [12]. A possible explanation is that the NHI program only reimbursed for lipid-lowering agents among patients who had severe hyperlipidemia or who were comorbid with other cardiometabolic disease. Physicians might provide health information for those with mild dyslipidemia but not code the diagnosis if they did not prescribe lipid-lowering agents. Furthermore, only the first three diagnostic codes for ambulatory claims would be included in the NHIRD. Patients with dyslipidemia also usually have other serious comorbid conditions. Thus, the diagnosis of dyslipidemia might be coded in the original claims but not included in the NHIRD. The concordance of lipid-lowering agents was moderate, which is better than that for the diagnosis of dyslipidemia. Similar findings were also noted for osteoporosis, which was diagnosed via routine screening and patients were usually given lifestyle interventions initially. Thus, physicians might not code the diagnosis for most mild cases.

For composite diseases, such as renal diseases and heart diseases, the concordances were only fair. Lack of a specific definition of these clinical diagnoses might increase the discrepancy between self-reports and claims records. Of note, the prevalence of psychiatric illnesses based on self-reports was much lower than that based on claims records. A previous comprehensive survey using the Chinese-Modified Diagnostic Interview Schedule for psychiatric epidemiology found that the prevalence of major depressive disorder, bipolar disorder, and anxiety disorders was 1.14%, 0.17%, and 7.75%, respectively [13]. The estimated prevalence was similar to those based on claims records, rather than self-reports. Patients might lack insight or hesitate to disclose their psychiatric illness during a general survey. Thus, the concordance was only fair and the positive agreement was low.

The concordance of medication use was substantial, except for anti-asthmatic medications. Given that asthma attacks are episodic in nature, the use of anti-asthmatic medications would also be intermittent rather than regular. Therefore, patients might have difficulty recalling their exact use. The concordance of prescription records in the NHIRD was generally better and less varied than that of clinical diagnoses. Previous studies using different health claims databases also found that the concordance of medication use in claims records was better than that of other data sources [5], [14], [15].

We found that the concordance of health system utilization was generally substantial, which was compatible with a previous validity study of health system utilization [6]. In this study, we further explored the concordance of a preventive service, the routine health examination. We found that the concordance for a health examination was only moderate. Since a routine health examination by the NHI program is very convenient and can be performed in a clinical setting, participants might not even be aware that they received a routine health examination.

Individuals using private health insurance might under-estimate the prevalence of certain health conditions based on claims records. In Taiwan, approximately 64.8% have private health insurance [16]. However, almost all individuals with private health insurance are also enrolled in the NHI program. The role of private health insurance in Taiwan is only supplemental. Therefore, the effect of private health insurance on estimating prevalence-based claims records is mild.

We further conducted subgroup analyses for the concordance stratified by three different age groups. Generally, the concordance among participants aged 30–49 and ≧50 years were grossly consistent with the findings among the whole study sample. However, we found that the kappa values of most clinical diagnoses among participants aged between 12–29 years were relatively small. It should be noted that the prevalence of most examined diseases was quite low among this age group. Since kappa is sensitive to the prevalence of studied items, a low kappa might be attributed to the paradoxical effect of low prevalence.

### Strategies for improving the concordance

In this study, the prevalence of most clinical diagnoses based on self-reports was lower than that based on claims records, which was defined by the occurrence of one ambulatory or one inpatient claim. This could be overestimated because the physician might code a tentative diagnosis initially and change it later if further evidence did not support this initial diagnosis. Thus, using strict definitions would reduce the estimated prevalence based on claims records. However, whether the kappa could provide accurate estimated concordance would depend on the disease of interest. Generally, using strict definitions could improve the concordance in chronic and/or severe diseases such as diabetes, malignancy, and stroke. However, for diseases that are episodic in nature such as gout or asthma, using strict definitions would have no overt benefit on the concordance. For potentially underestimated diseases in the claims records, such as dyslipidemia and osteoporosis, using a broad definition to reflect both treated and untreated patients with these conditions might be better. As such, since there is no fixed algorithm which can be applied to all diseases, the use of strict or broad definitions should be cautiously considered based on the aims of the study and the trade-off between sensitivity and specificity [17].

### Factors associated with disconcordance

In our analysis of the participant characteristics associated with disconcordance, we found that age has an important impact on disconcordance in diseases and medication use. Compared with young adults, the elderly have more complex diseases and a greater vulnerability to memory impairment; therefore, their self-reports are more likely to be discrepant with the claims records. These findings are consistent with previous studies showing that those at an older age or with a complex comorbidity were more likely to have discordance between self-reports and claims records [18], [19]. In addition, a low level of education was related to poor health knowledge and physician-patient communication, and thereby had a negative effect on the agreement between self-reports and claims records [20]. Single status was associated with more actual agreement. In Taiwan, patient family members actively participate in medical treatment decision-making. If the patient has a serious disease, such as a malignancy, the physician might discuss this with family members first, before the patient himself/herself. The family members might even decide to conceal this information to avoid possible negative emotional reactions of the patient.

To explore the effect of disease-specific factors on the concordance in clinical diagnosis, we assessed the effect of obesity on the concordance in hypertension, diabetes mellitus, and dyslipidemia. Obesity is strongly associated with these cardiometabolic disorders but not with psychiatric illness. While we found that obesity was associated with disagreement between claims records and self-reports in hypertension, diabetes mellitus, and dyslipidemia, obesity was not related to that in psychiatric illness. A possible explanation is that patients with obesity are more likely to fall in a gray area of definitive diagnosis; therefore, they might be confused about whether or not they have obesity-related cardiometabolic disorders. In contrast, the effect of obesity on the concordance in psychiatric illness was not significant. In summary, patients with strong risk factors for cardiometabolic disorders might increase the proportion of disagreement between claims record and self-reports.

### Validity of the NHIRD and NHIS

Despite a dramatic increase in the publication of scientific papers using the NHIRD, the validity of NHIRD is still under-investigated. One study conducted a medical chart review of 372 stroke patients and found that the accuracy of the discharge diagnosis for ischemic stroke in the NHIRD was high; in particular, the positive predictive value was 97.9% [21]. Another study reviewed 354 randomly selected medical charts and found that the accuracy of the discharge diagnoses of acute coronary syndrome was 100% [22]. In addition, Lin et al. looked at patient self-report on a mailed-in questionnaire and found that the rate of agreement with a diabetes diagnosis in either ambulatory or hospital claims was 74.6% [8]. To date, only one study validated the self-reported health services utilization of the NHIS by using the claims records as reference standards [6]. Our study explored the concordance between the NHIS and NHIRD and provided important information regarding the validity of both data sources. Further validation studies of the NHIRD and NHIS should focus on specific conditions and develop optimal definitions based on the nature of the clinical conditions being assessed and the aims of the study.

### Comparison of the concordance of self-reports and claims records in other claims data

We found that the concordance of the NHIRD was comparable to that of other claims databases throughout the world. One study explored the concordance of hypertension diagnoses between a patient survey and an insurance claims database in the U.S. and found that the proportion of agreement was 0.96 [23]. In addition, the kappa statistic for hypertension between the Manitoba Heart Health Survey and physician service claims files was 0.56 [18]. These findings are similar to our findings for hypertension (the total agreement was 0.93; kappa was 0.69). In terms of the concordance of diabetes diagnoses, the kappa statistic between self-reports and claims records was 0.74 in U.S Medicare claims [24] and 0.80 in Medicare Australian data [25]. The kappa statistics of medication use between self-reports and claims data ranged from 0.69 to 0.80, depending on the type of medication use [14], [26], [27].

### Limitations and strengths of the study

Several limitations for this study should be considered. First, the date of interview was not available in the 2005 Taiwan NHIS. As such, we used the approximate median date of the survey as the date of interview for all participants. Since the study timeframe could not be more precisely defined. Lack of an exact timeframe would underestimate the degree of concordance and the proportion of agreement, especially for those conditions that are episodic in nature. Second, 25.2% of participants, or those who did consent to link the NHIRD, were not included in this study. Compared with consenters, those excluded from the analysis were slightly elder and less educated, and were less likely to be living in an urban area, which were characteristics that were associated with high disconcordance. [6], [18], [20] Thus, the concordance between self-reports and claims records might be overestimated. Third, since disease-specific factors could provide more information regarding each examined disease, we further examined the effect of obesity on hypertension, diabetes, dyslipidemia, and psychiatric disorders, respectively. However, we did not have additional collected data for assessing disease-specific factors for the other examined diseases. Further investigation on disease-specific factors for these other examined diseases is warranted.

Despite the above-mentioned limitations, this is the first study to pervasively and comprehensively assess the concordance between NHIRD claims records and a nationwide representative survey on clinical diagnosis and medication use. Given that details regarding socio-demographic and lifestyle data in the NHIRD and comprehensive information on medication exposure and procedures in the NHIS were not available, the linkage between the NHIS and NHIRD could complement the strength of each system, and enhance the utilization of both data sources in epidemiology and health services research.

## Conclusions

The overall concordance between the claims records and self-report survey results was moderate in clinical diagnoses. However, the concordance varied markedly across different diagnoses. In summary, the concordance in common chronic disease was substantial. The concordance in severe diseases was moderate, which might be attributable to low prevalence. Regarding diseases with episodic nature, as well as composite diseases without clear definitions, the concordance was only fair. In terms of medication use and health system utilization, the concordance was substantial. In terms of research implications, the linkage between the NHIS and NHIRD could enhance the utilization of both datasets.

## Supporting Information

Table S1 **Claims records for clinical diagnoses and medication use in the NHIRD.**
(DOC)

Table S2 **Concordance between self-report and claims record, by diagnoses, medication use, and health system utilization among participants aged 12–29 years.**
(DOC)

Table S3 **Concordance between self-report and claims record, by diagnoses, medication use, and health system utilization among participants aged 30–49 years.**
(DOC)

Table S4 **Concordance between self-report and claims record, by diagnoses, medication use, and health system utilization among participants aged 50 years and older.**
(DOC)

Table S5 **Factors associated with disagreement between self-report and claims record in hypertension, diabetes mellitus, dyslipidemia, and psychiatric disorders.**
(DOC)
