# Supplementary material for: Concordance between Patient Self-Reports and Claims Data on Clinical Diagnoses, Medication Use, and Health System Utilization in Taiwan
Source: PLoS One. 2014 Dec 2;9(12):e112257. doi: 10.1371/journal.pone.0112257 (PMC4251897; doi:10.1371/journal.pone.0112257)
Supplement: Table S1 — Claims records for clinical diagnoses and medication use in the NHIRD. (DOC) [file pone.0112257.s001.doc]

Supplemental Table S1. Claims records for clinical diagnoses and medication use in the NHIRD.

| Condition | |  | Claims records |
| --- | --- | --- | --- |
| **Clinical diagnoses** | | **Composite diseases** | **ICD-9CM code** |
|  | Hypertension |  | 401-405 |
|  | Diabetes mellitus |  | 250 |
|  | Dyslipidemia |  | 272 |
|  | Stroke |  | 430-438 |
|  | Asthma^†^ |  | 493 |
|  | Renal disease^†^ |  | 580-593 |
|  | Heart disease^†^ | Rheumatic heart disease, valvular heart disease, hypertensive heart disease, angina pectoris, ischemic heart disease, myocardial infarction, or arrhythmia | 393-398, 402, 404, 410-414, 426, 427 |
|  | Gout^†^ |  | 274 |
|  | Chronic pulmonary disease^†^ | Emphysema, chronic bronchitis, bronchiectasis, or chronic airway obstruction | 490-492, 494, 496 |
|  | Osteoporosis^†^ |  | 733.0 |
|  | Chronic hepatitis or liver cirrhosis^†^ |  | 571, 070 |
|  | Cancer^†^ | Leukemia, malignancy of liver, lung, colon and sigmoid, oral, gastric, prostate, bladder, esophageal, skin, nasopharynx, cervical, breast, thyroid, ovary, and uterus | 140-155, 162, 164, 172-175, 180, 182, 183, 185, 188, 200-208 |
|  | Psychiatric disorders^†^ | Depressive disorder, bipolar disorders, or anxiety disorders | 296, 300, 311 |
|  | Arthritis^†^ |  | 714, 715 |
| **Medication use** | |  | **ATC-code** |
|  | Anti-hypertensive agent | | C03, C07, C08, C09 |
|  | Anti-diabetic agent |  | A10 |
|  | Lipid-lowering agent | | C10 |
|  | Anti-asthma agent |  | R03 |
|  | Anti-gout agent |  | M04 |

Note:

^†^ Patients self-reporting a disease status were asked for a timeframe "Did you have this disease during the last year?”

Supplemental Table S2. Concordance between self-report and claims record, by diagnoses, medication use, and health system utilization among participants aged 12-29 years

|  |  | Self-reports (%) | Claims records (%) | In claims records, in self-reports (%) | In self-reports only (%) | In claims records only (%) | Not in claims records, not in self-reports (%) | Total agreement | Positive agreement | Negative agreement | Kappa |
| --- | --- | --- | --- | --- | --- | --- | --- | --- | --- | --- | --- |
| **Diagnoses** | |  |  |  |  |  |  |  |  |  |  |
|  | Hypertension | 0.8 | 0.4 | 0.1 | 0.6 | 0.3 | 98.9 | 0.99 | 0.19 | 1.00 | 0.19 |
|  | Diabetes | 0.3 | 0.3 | 0.1 | 0.1 | 0.2 | 99.6 | 1.00 | 0.41 | 1.00 | 0.41 |
|  | Dyslipidemia | 2.5 | 0.9 | 0.3 | 2.2 | 0.6 | 97.0 | 0.97 | 0.18 | 0.99 | 0.17 |
|  | Malignancy | 0.1 | 0.1 | 0.0 | 0.1 | 0.0 | 99.9 | 1.00 | 0.25 | 1.00 | 0.25 |
|  | Stroke | 0.0 | 0.1 | 0.0 | 0.0 | 0.1 | 99.9 | 1.00 | 0.00 | 1.00 | 0.00 |
|  | Asthma | 1.8 | 1.7 | 0.7 | 1.2 | 1.0 | 97.2 | 0.98 | 0.38 | 0.99 | 0.37 |
|  | Chronic pulmonary diseases | 1.4 | 2.1 | 0.2 | 1.3 | 1.9 | 96.6 | 0.97 | 0.09 | 0.98 | 0.08 |
|  | Gout | 0.8 | 1.3 | 0.4 | 0.4 | 0.8 | 98.4 | 0.99 | 0.42 | 0.99 | 0.42 |
|  | Osteoporosis | 0.0 | 0.1 | 0.0 | 0.0 | 0.1 | 99.9 | 1.00 | 0.00 | 1.00 | 0.00 |
|  | Arthritis | 0.4 | 0.9 | 0.1 | 0.3 | 0.8 | 98.8 | 0.99 | 0.12 | 0.99 | 0.11 |
|  | Renal diseases | 1.0 | 1.1 | 0.2 | 0.7 | 0.9 | 98.2 | 0.98 | 0.21 | 0.99 | 0.21 |
|  | Heart diseases | 1.0 | 1.0 | 0.3 | 0.7 | 0.7 | 98.3 | 0.99 | 0.27 | 0.99 | 0.26 |
|  | Chronic hepatitis | 1.5 | 3.0 | 0.8 | 0.7 | 2.2 | 96.3 | 0.97 | 0.36 | 0.99 | 0.35 |
|  | Psychiatric disorders | 0.7 | 2.2 | 0.4 | 0.4 | 1.8 | 97.5 | 0.98 | 0.24 | 0.99 | 0.23 |
|  | Overall | 0.9 | 1.1 | 0.3 | 0.6 | 0.8 | 98.3 | 0.99 | 0.26 | 0.99 | 0.25 |
| **Medication use** | |  |  |  |  |  |  |  |  |  |  |
|  | Anti-hypertensives | 0.2 | 0.2 | 0.1 | 0.2 | 0.2 | 99.6 | 1.00 | 0.31 | 1.00 | 0.31 |
|  | Anti-diabetes | 0.1 | 0.1 | 0.1 | 0.0 | 0.0 | 99.8 | 1.00 | 0.80 | 1.00 | 0.80 |
|  | Lipid lowering agents | 0.1 | 0.1 | 0.0 | 0.1 | 0.0 | 99.9 | 1.00 | 0.40 | 1.00 | 0.40 |
|  | Anti-asthmatics | 0.9 | 1.4 | 0.5 | 0.4 | 0.9 | 98.1 | 0.99 | 0.42 | 0.99 | 0.41 |
|  | Anti-gout drugs | 0.6 | 0.7 | 0.3 | 0.3 | 0.5 | 99.0 | 0.99 | 0.41 | 1.00 | 0.40 |
|  | Overall | 0.4 | 0.5 | 0.2 | 0.2 | 0.3 | 99.3 | 0.99 | 0.43 | 1.00 | 0.42 |
| **Health system utilization** | |  |  |  |  |  |  |  |  |  |  |
|  | Hospitalization | 3.6 | 5.2 | 2.3 | 1.3 | 2.9 | 93.5 | 0.96 | 0.53 | 0.98 | 0.51 |
|  | Emergence room visit | 12.9 | 17.7 | 9.3 | 3.6 | 8.5 | 78.7 | 0.88 | 0.61 | 0.93 | 0.54 |
|  | Dentistry services | 40.1 | 39.1 | 29.5 | 10.6 | 9.6 | 50.3 | 0.80 | 0.74 | 0.83 | 0.58 |
|  | Health examination | 0.2 | 0.0 | 0.0 | 0.2 | 0.0 | 99.8 | 1.00 | 0.00 | 1.00 | 0.00 |
|  | Overall | 14.2 | 15.5 | 10.3 | 3.9 | 5.2 | 80.6 | 0.91 | 0.69 | 0.95 | 0.64 |

Supplemental Table S3. Concordance between self-report and claims record, by diagnoses, medication use, and health system utilization among participants aged 30-49 years

|  |  | Self-reports (%) | Claims records (%) | In claims records, in self-reports (%) | In self-reports only (%) | In claims records only (%) | Not in claims records, not in self-reports (%) | Total agreement | Positive agreement | Negative agreement | Kappa |
| --- | --- | --- | --- | --- | --- | --- | --- | --- | --- | --- | --- |
| **Diagnoses** | |  |  |  |  |  |  |  |  |  |  |
|  | Hypertension | 6.3 | 5.7 | 3.9 | 2.4 | 1.9 | 91.9 | 0.96 | 0.64 | 0.98 | 0.62 |
|  | Diabetes | 2.1 | 2.7 | 1.6 | 0.5 | 1.1 | 96.8 | 0.98 | 0.67 | 0.99 | 0.67 |
|  | Dyslipidemia | 11.7 | 4.8 | 2.5 | 9.2 | 2.3 | 86.0 | 0.89 | 0.30 | 0.94 | 0.25 |
|  | Malignancy | 0.7 | 1.2 | 0.5 | 0.2 | 0.6 | 98.7 | 0.99 | 0.58 | 1.00 | 0.58 |
|  | Stroke | 0.2 | 0.7 | 0.1 | 0.1 | 0.5 | 99.3 | 0.99 | 0.33 | 1.00 | 0.33 |
|  | Asthma | 1.1 | 2.1 | 0.5 | 0.6 | 1.6 | 97.3 | 0.98 | 0.32 | 0.99 | 0.31 |
|  | Chronic pulmonary diseases | 1.6 | 3.3 | 0.2 | 1.4 | 3.1 | 95.3 | 0.96 | 0.09 | 0.98 | 0.07 |
|  | Gout | 3.6 | 4.2 | 2.3 | 1.3 | 1.9 | 94.5 | 0.97 | 0.59 | 0.98 | 0.57 |
|  | Osteoporosis | 1.4 | 0.4 | 0.1 | 1.3 | 0.3 | 98.4 | 0.98 | 0.10 | 0.99 | 0.10 |
|  | Arthritis | 1.7 | 3.9 | 0.6 | 1.1 | 3.3 | 95.0 | 0.96 | 0.21 | 0.98 | 0.19 |
|  | Renal diseases | 3.5 | 3.8 | 1.1 | 2.5 | 2.7 | 93.8 | 0.95 | 0.29 | 0.97 | 0.26 |
|  | Heart diseases | 1.7 | 3.3 | 0.8 | 0.9 | 2.5 | 95.8 | 0.97 | 0.33 | 0.98 | 0.32 |
|  | Chronic hepatitis | 3.7 | 7.8 | 2.3 | 1.5 | 5.6 | 90.7 | 0.93 | 0.39 | 0.96 | 0.36 |
|  | Psychiatric disorders | 2.2 | 6.8 | 1.4 | 0.8 | 5.4 | 92.5 | 0.94 | 0.31 | 0.97 | 0.29 |
|  | Overall | 3.0 | 3.6 | 1.3 | 1.7 | 2.3 | 94.7 | 0.96 | 0.39 | 0.98 | 0.37 |
| **Medication use** | |  |  |  |  |  |  |  |  |  |  |
|  | Anti-hypertensives | 4.3 | 5.0 | 3.3 | 1.0 | 1.7 | 94.0 | 0.97 | 0.71 | 0.99 | 0.69 |
|  | Anti-diabetes | 1.7 | 1.8 | 1.5 | 0.2 | 0.3 | 98.0 | 0.99 | 0.85 | 1.00 | 0.85 |
|  | Lipid lowering agents | 2.4 | 1.9 | 1.1 | 1.3 | 0.8 | 96.8 | 0.98 | 0.50 | 0.99 | 0.49 |
|  | Anti-asthmatics | 0.7 | 1.7 | 0.4 | 0.3 | 1.3 | 98.0 | 0.98 | 0.34 | 0.99 | 0.33 |
|  | Anti-gout drugs | 2.8 | 3.3 | 1.6 | 1.2 | 1.7 | 95.5 | 0.97 | 0.52 | 0.98 | 0.50 |
|  | Overall | 2.4 | 2.8 | 1.6 | 0.8 | 1.2 | 96.5 | 0.98 | 0.61 | 0.99 | 0.60 |
| **Health system utilization** | |  |  |  |  |  |  |  |  |  |  |
|  | Hospitalization | 5.7 | 6.7 | 4.2 | 1.5 | 2.5 | 91.8 | 0.96 | 0.67 | 0.98 | 0.65 |
|  | Emergence room visit | 12.0 | 13.8 | 7.9 | 4.1 | 5.9 | 82.1 | 0.90 | 0.61 | 0.94 | 0.56 |
|  | Dentistry services | 38.8 | 36.4 | 28.5 | 10.2 | 7.9 | 53.3 | 0.82 | 0.76 | 0.85 | 0.61 |
|  | Health examination | 4.4 | 5.5 | 1.9 | 2.5 | 3.5 | 92.0 | 0.94 | 0.39 | 0.97 | 0.36 |
|  | Overall | 15.2 | 15.6 | 10.6 | 4.6 | 5.0 | 79.8 | 0.90 | 0.69 | 0.94 | 0.63 |

Supplemental Table S4. Concordance between self-report and claims record, by diagnoses, medication use, and health system utilization among participants aged 50 years and older

|  |  | Self-reports (%) | Claims records (%) | In claims records, in self-reports (%) | In self-reports only (%) | In claims records only (%) | Not in claims records, not in self-reports (%) | Total agreement | Positive agreement | Negative agreement | Kappa |
| --- | --- | --- | --- | --- | --- | --- | --- | --- | --- | --- | --- |
| **Diagnoses** | |  |  |  |  |  |  |  |  |  |  |
|  | Hypertension | 31.5 | 36.3 | 25.7 | 5.7 | 10.5 | 58.0 | 0.84 | 0.76 | 0.88 | 0.64 |
|  | Diabetes | 13.4 | 16.2 | 11.8 | 1.6 | 4.4 | 82.2 | 0.94 | 0.80 | 0.96 | 0.76 |
|  | Dyslipidemia | 22.4 | 16.7 | 8.7 | 13.7 | 8.0 | 69.6 | 0.78 | 0.45 | 0.87 | 0.31 |
|  | Malignancy | 1.9 | 4.0 | 1.7 | 0.2 | 2.3 | 95.8 | 0.97 | 0.58 | 0.99 | 0.56 |
|  | Stroke | 3.9 | 7.9 | 2.7 | 1.3 | 5.2 | 90.8 | 0.93 | 0.45 | 0.97 | 0.42 |
|  | Asthma | 3.6 | 5.2 | 1.8 | 1.8 | 3.4 | 93.0 | 0.95 | 0.41 | 0.97 | 0.39 |
|  | Chronic pulmonary diseases | 4.6 | 11.6 | 2.2 | 2.3 | 9.4 | 86.0 | 0.88 | 0.28 | 0.94 | 0.22 |
|  | Gout | 6.7 | 7.9 | 3.3 | 3.4 | 4.6 | 88.7 | 0.92 | 0.45 | 0.96 | 0.41 |
|  | Osteoporosis | 11.1 | 6.1 | 2.7 | 8.4 | 3.4 | 85.5 | 0.88 | 0.31 | 0.94 | 0.25 |
|  | Arthritis | 8.9 | 18.4 | 4.7 | 4.2 | 13.7 | 77.4 | 0.82 | 0.35 | 0.90 | 0.26 |
|  | Renal diseases | 7.0 | 8.0 | 3.0 | 4.0 | 5.0 | 88.0 | 0.91 | 0.40 | 0.95 | 0.35 |
|  | Heart diseases | 10.4 | 23.1 | 7.7 | 2.6 | 15.4 | 74.3 | 0.82 | 0.46 | 0.89 | 0.37 |
|  | Chronic hepatitis | 4.7 | 11.1 | 3.1 | 1.6 | 7.9 | 87.4 | 0.91 | 0.40 | 0.95 | 0.36 |
|  | Psychiatric disorders | 2.0 | 12.3 | 1.1 | 0.8 | 11.2 | 86.8 | 0.88 | 0.16 | 0.94 | 0.13 |
|  | Overall | 9.4 | 13.2 | 5.7 | 3.7 | 7.5 | 83.1 | 0.89 | 0.51 | 0.94 | 0.45 |
| **Medication use** | |  |  |  |  |  |  |  |  |  |  |
|  | Anti-hypertensives | 28.0 | 32.5 | 23.0 | 5.0 | 9.5 | 62.5 | 0.85 | 0.76 | 0.90 | 0.66 |
|  | Anti-diabetes | 12.1 | 12.8 | 10.6 | 1.5 | 2.2 | 85.8 | 0.96 | 0.85 | 0.98 | 0.83 |
|  | Lipid lowering agents | 9.6 | 8.7 | 4.1 | 5.5 | 4.6 | 85.8 | 0.90 | 0.45 | 0.94 | 0.39 |
|  | Anti-asthmatics | 3.0 | 4.5 | 1.5 | 1.5 | 3.0 | 94.0 | 0.95 | 0.40 | 0.98 | 0.38 |
|  | Anti-gout drugs | 6.1 | 5.5 | 2.4 | 3.6 | 3.1 | 90.8 | 0.93 | 0.42 | 0.96 | 0.38 |
|  | Overall | 11.8 | 12.8 | 8.3 | 3.4 | 4.5 | 83.8 | 0.92 | 0.68 | 0.95 | 0.63 |
| **Health system utilization** | |  |  |  |  |  |  |  |  |  |  |
|  | Hospitalization | 12.8 | 12.9 | 9.9 | 2.8 | 3.0 | 84.3 | 0.94 | 0.77 | 0.97 | 0.74 |
|  | Emergence room visit | 15.9 | 18.2 | 10.4 | 5.5 | 7.8 | 76.4 | 0.87 | 0.61 | 0.92 | 0.53 |
|  | Dentistry services | 36.1 | 35.8 | 26.6 | 9.6 | 9.2 | 54.6 | 0.81 | 0.74 | 0.85 | 0.59 |
|  | Health examination | 17.4 | 20.9 | 8.7 | 8.8 | 12.3 | 70.3 | 0.79 | 0.45 | 0.87 | 0.32 |
|  | Overall | 20.5 | 22.0 | 13.9 | 6.7 | 8.1 | 71.4 | 0.85 | 0.65 | 0.91 | 0.56 |

Supplemental Table S5. Factors associated with disagreement between self-report and claims record in hypertension, diabetes mellitus, dyslipidemia, and psychiatric disorders

|  |  | Hypertension | |  | Diabetes |  |  | Dyslipidemia | |  | Psychiatric disorders | | |  |  |  |  |  |  |
| --- | --- | --- | --- | --- | --- | --- | --- | --- | --- | --- | --- | --- | --- | --- | --- | --- | --- | --- | --- |
|  |  | Odds ratio | (95% CI) | | Odds ratio | (95% CI) | | Odds ratio | (95% CI) | | Odds ratio | (95% CI) | |  |  |  |  |  |  |
| Age group (vs. 12-29) | |  |  |  |  |  |  |  |  |  |  |  |  |  |  |  |  |  |  |
| 30-49 |  | 4.43 | (3.07 | , 6.40) | 3.43 | (1.82 | , 6.47) | 2.80 | (2.21 | , 3.56) | 2.68 | (2.01 | , 3.56) |  |  |  |  |  |  |
| ≥50 |  | 15.13 | (10.28 | , 22.27) | 10.62 | (5.50 | , 20.50) | 5.63 | (4.35 | , 7.28) | 4.77 | (3.48 | , 6.54) |  |  |  |  |  |  |
| Gender |  |  |  |  |  |  |  |  |  |  |  |  |  |  |  |  |  |  |  |
| Male vs. female | | 1.27 | (1.11 | , 1.46) | 1.11 | (0.90 | , 1.39) | 1.08 | (0.97 | , 1.21) | 0.65 | (0.57 | , 0.75) |  |  |  |  |  |  |
| Education (vs. ≤6 years)^a^ | |  |  |  |  |  |  |  |  |  |  |  |  |  |  |  |  |  |  |
| 7-12 |  | 0.64 | (0.53 | , 0.76) | 0.70 | (0.53 | , 0.91) | 0.81 | (0.70 | , 0.93) | 0.79 | (0.66 | , 0.95) |  |  |  |  |  |  |
| ≥13 |  | 0.65 | (0.52 | , 0.81) | 0.60 | (0.42 | , 0.86) | 1.13 | (0.96 | , 1.34) | 0.66 | (0.53 | , 0.82) |  |  |  |  |  |  |
| Marriage^a^ (vs. single) | |  |  |  |  |  |  |  |  |  |  |  |  |  |  |  |  |  |  |
| Married/living as married | | 1.00 | (0.75 | , 1.32) | 1.39 | (0.85 | , 2.29) | 1.67 | (1.35 | , 2.06) | 1.07 | (0.83 | , 1.38) |  |  |  |  |  |  |
| Divorced/widowed/separated | | 0.84 | (0.58 | , 1.21) | 1.15 | (0.62 | , 2.14) | 1.40 | (1.05 | , 1.86) | 1.16 | (0.83 | , 1.63) |  |  |  |  |  |  |
| Residence (vs. urban) | |  |  |  |  |  |  |  |  |  |  |  |  |  |  |  |  |  |  |
| Sub-urban | | 1.13 | (0.97 | , 1.31) | 1.00 | (0.79 | , 1.27) | 0.89 | (0.80 | , 1.00) | 0.92 | (0.79 | , 1.06) |  |  |  |  |  |  |
| Rural |  | 1.14 | (0.94 | , 1.38) | 1.16 | (0.87 | , 1.55) | 0.91 | (0.78 | , 1.06) | 1.01 | (0.84 | , 1.22) |  |  |  |  |  |  |
| Body mass index (vs. <24) | |  |  |  |  |  |  |  |  |  |  |  |  |  |  |  |  |  |  |
| Overweight (BMI 24-26 | | 0.99 | (0.84 | , 1.18) | 1.34 | (1.02 | , 1.76) | 1.79 | (1.57 | , 2.04) | 0.99 | (0.84 | , 1.16) |  |  |  |  |  |  |
| Obesity (BMI ≥ 27) | | 1.63 | (1.40 | , 1.91) | 2.25 | (1.76 | , 2.88) | 2.58 | (2.28 | , 2.93) | 0.95 | (0.80 | , 1.13) |  |  |  |  |  |  |

Note:

^a^ Significance was determined using the multivariate generalized estimating equation by Wald Z test with df = 1 and was declared as *p*<0.05.
