# Supplementary material for: Concordance between Patient Self-Reports and Claims Data on Clinical Diagnoses, Medication Use, and Health System Utilization in Taiwan
Source: PLoS One. 2014 Dec 2;9(12):e112257. doi: 10.1371/journal.pone.0112257 (PMC4251897; doi:10.1371/journal.pone.0112257)
Supplement: Table S2 — Concordance between self-report and claims record, by diagnoses, medication use, and health system utilization among participants aged 12–29 years. (DOC) [file pone.0112257.s002.doc]

Supplemental Table S2. Concordance between self-report and claims record, by diagnoses, medication use, and health system utilization among participants aged 12-29 years

|  |  | Self-reports (%) | Claims records (%) | In claims records, in self-reports (%) | In self-reports only (%) | In claims records only (%) | Not in claims records, not in self-reports (%) | Total agreement | Positive agreement | Negative agreement | Kappa |
| --- | --- | --- | --- | --- | --- | --- | --- | --- | --- | --- | --- |
| **Diagnoses** | |  |  |  |  |  |  |  |  |  |  |
|  | Hypertension | 0.8 | 0.4 | 0.1 | 0.6 | 0.3 | 98.9 | 0.99 | 0.19 | 1.00 | 0.19 |
|  | Diabetes | 0.3 | 0.3 | 0.1 | 0.1 | 0.2 | 99.6 | 1.00 | 0.41 | 1.00 | 0.41 |
|  | Dyslipidemia | 2.5 | 0.9 | 0.3 | 2.2 | 0.6 | 97.0 | 0.97 | 0.18 | 0.99 | 0.17 |
|  | Malignancy | 0.1 | 0.1 | 0.0 | 0.1 | 0.0 | 99.9 | 1.00 | 0.25 | 1.00 | 0.25 |
|  | Stroke | 0.0 | 0.1 | 0.0 | 0.0 | 0.1 | 99.9 | 1.00 | 0.00 | 1.00 | 0.00 |
|  | Asthma | 1.8 | 1.7 | 0.7 | 1.2 | 1.0 | 97.2 | 0.98 | 0.38 | 0.99 | 0.37 |
|  | Chronic pulmonary diseases | 1.4 | 2.1 | 0.2 | 1.3 | 1.9 | 96.6 | 0.97 | 0.09 | 0.98 | 0.08 |
|  | Gout | 0.8 | 1.3 | 0.4 | 0.4 | 0.8 | 98.4 | 0.99 | 0.42 | 0.99 | 0.42 |
|  | Osteoporosis | 0.0 | 0.1 | 0.0 | 0.0 | 0.1 | 99.9 | 1.00 | 0.00 | 1.00 | 0.00 |
|  | Arthritis | 0.4 | 0.9 | 0.1 | 0.3 | 0.8 | 98.8 | 0.99 | 0.12 | 0.99 | 0.11 |
|  | Renal diseases | 1.0 | 1.1 | 0.2 | 0.7 | 0.9 | 98.2 | 0.98 | 0.21 | 0.99 | 0.21 |
|  | Heart diseases | 1.0 | 1.0 | 0.3 | 0.7 | 0.7 | 98.3 | 0.99 | 0.27 | 0.99 | 0.26 |
|  | Chronic hepatitis | 1.5 | 3.0 | 0.8 | 0.7 | 2.2 | 96.3 | 0.97 | 0.36 | 0.99 | 0.35 |
|  | Psychiatric disorders | 0.7 | 2.2 | 0.4 | 0.4 | 1.8 | 97.5 | 0.98 | 0.24 | 0.99 | 0.23 |
|  | Overall | 0.9 | 1.1 | 0.3 | 0.6 | 0.8 | 98.3 | 0.99 | 0.26 | 0.99 | 0.25 |
| **Medication use** | |  |  |  |  |  |  |  |  |  |  |
|  | Anti-hypertensives | 0.2 | 0.2 | 0.1 | 0.2 | 0.2 | 99.6 | 1.00 | 0.31 | 1.00 | 0.31 |
|  | Anti-diabetes | 0.1 | 0.1 | 0.1 | 0.0 | 0.0 | 99.8 | 1.00 | 0.80 | 1.00 | 0.80 |
|  | Lipid lowering agents | 0.1 | 0.1 | 0.0 | 0.1 | 0.0 | 99.9 | 1.00 | 0.40 | 1.00 | 0.40 |
|  | Anti-asthmatics | 0.9 | 1.4 | 0.5 | 0.4 | 0.9 | 98.1 | 0.99 | 0.42 | 0.99 | 0.41 |
|  | Anti-gout drugs | 0.6 | 0.7 | 0.3 | 0.3 | 0.5 | 99.0 | 0.99 | 0.41 | 1.00 | 0.40 |
|  | Overall | 0.4 | 0.5 | 0.2 | 0.2 | 0.3 | 99.3 | 0.99 | 0.43 | 1.00 | 0.42 |
| **Health system utilization** | |  |  |  |  |  |  |  |  |  |  |
|  | Hospitalization | 3.6 | 5.2 | 2.3 | 1.3 | 2.9 | 93.5 | 0.96 | 0.53 | 0.98 | 0.51 |
|  | Emergence room visit | 12.9 | 17.7 | 9.3 | 3.6 | 8.5 | 78.7 | 0.88 | 0.61 | 0.93 | 0.54 |
|  | Dentistry services | 40.1 | 39.1 | 29.5 | 10.6 | 9.6 | 50.3 | 0.80 | 0.74 | 0.83 | 0.58 |
|  | Health examination | 0.2 | 0.0 | 0.0 | 0.2 | 0.0 | 99.8 | 1.00 | 0.00 | 1.00 | 0.00 |
|  | Overall | 14.2 | 15.5 | 10.3 | 3.9 | 5.2 | 80.6 | 0.91 | 0.69 | 0.95 | 0.64 |
