# Supplementary material for: Concordance between Patient Self-Reports and Claims Data on Clinical Diagnoses, Medication Use, and Health System Utilization in Taiwan
Source: PLoS One. 2014 Dec 2;9(12):e112257. doi: 10.1371/journal.pone.0112257 (PMC4251897; doi:10.1371/journal.pone.0112257)
Supplement: Table S3 — Concordance between self-report and claims record, by diagnoses, medication use, and health system utilization among participants aged 30–49 years. (DOC) [file pone.0112257.s003.doc]

Supplemental Table S3. Concordance between self-report and claims record, by diagnoses, medication use, and health system utilization among participants aged 30-49 years

|  |  | Self-reports (%) | Claims records (%) | In claims records, in self-reports (%) | In self-reports only (%) | In claims records only (%) | Not in claims records, not in self-reports (%) | Total agreement | Positive agreement | Negative agreement | Kappa |
| --- | --- | --- | --- | --- | --- | --- | --- | --- | --- | --- | --- |
| **Diagnoses** | |  |  |  |  |  |  |  |  |  |  |
|  | Hypertension | 6.3 | 5.7 | 3.9 | 2.4 | 1.9 | 91.9 | 0.96 | 0.64 | 0.98 | 0.62 |
|  | Diabetes | 2.1 | 2.7 | 1.6 | 0.5 | 1.1 | 96.8 | 0.98 | 0.67 | 0.99 | 0.67 |
|  | Dyslipidemia | 11.7 | 4.8 | 2.5 | 9.2 | 2.3 | 86.0 | 0.89 | 0.30 | 0.94 | 0.25 |
|  | Malignancy | 0.7 | 1.2 | 0.5 | 0.2 | 0.6 | 98.7 | 0.99 | 0.58 | 1.00 | 0.58 |
|  | Stroke | 0.2 | 0.7 | 0.1 | 0.1 | 0.5 | 99.3 | 0.99 | 0.33 | 1.00 | 0.33 |
|  | Asthma | 1.1 | 2.1 | 0.5 | 0.6 | 1.6 | 97.3 | 0.98 | 0.32 | 0.99 | 0.31 |
|  | Chronic pulmonary diseases | 1.6 | 3.3 | 0.2 | 1.4 | 3.1 | 95.3 | 0.96 | 0.09 | 0.98 | 0.07 |
|  | Gout | 3.6 | 4.2 | 2.3 | 1.3 | 1.9 | 94.5 | 0.97 | 0.59 | 0.98 | 0.57 |
|  | Osteoporosis | 1.4 | 0.4 | 0.1 | 1.3 | 0.3 | 98.4 | 0.98 | 0.10 | 0.99 | 0.10 |
|  | Arthritis | 1.7 | 3.9 | 0.6 | 1.1 | 3.3 | 95.0 | 0.96 | 0.21 | 0.98 | 0.19 |
|  | Renal diseases | 3.5 | 3.8 | 1.1 | 2.5 | 2.7 | 93.8 | 0.95 | 0.29 | 0.97 | 0.26 |
|  | Heart diseases | 1.7 | 3.3 | 0.8 | 0.9 | 2.5 | 95.8 | 0.97 | 0.33 | 0.98 | 0.32 |
|  | Chronic hepatitis | 3.7 | 7.8 | 2.3 | 1.5 | 5.6 | 90.7 | 0.93 | 0.39 | 0.96 | 0.36 |
|  | Psychiatric disorders | 2.2 | 6.8 | 1.4 | 0.8 | 5.4 | 92.5 | 0.94 | 0.31 | 0.97 | 0.29 |
|  | Overall | 3.0 | 3.6 | 1.3 | 1.7 | 2.3 | 94.7 | 0.96 | 0.39 | 0.98 | 0.37 |
| **Medication use** | |  |  |  |  |  |  |  |  |  |  |
|  | Anti-hypertensives | 4.3 | 5.0 | 3.3 | 1.0 | 1.7 | 94.0 | 0.97 | 0.71 | 0.99 | 0.69 |
|  | Anti-diabetes | 1.7 | 1.8 | 1.5 | 0.2 | 0.3 | 98.0 | 0.99 | 0.85 | 1.00 | 0.85 |
|  | Lipid lowering agents | 2.4 | 1.9 | 1.1 | 1.3 | 0.8 | 96.8 | 0.98 | 0.50 | 0.99 | 0.49 |
|  | Anti-asthmatics | 0.7 | 1.7 | 0.4 | 0.3 | 1.3 | 98.0 | 0.98 | 0.34 | 0.99 | 0.33 |
|  | Anti-gout drugs | 2.8 | 3.3 | 1.6 | 1.2 | 1.7 | 95.5 | 0.97 | 0.52 | 0.98 | 0.50 |
|  | Overall | 2.4 | 2.8 | 1.6 | 0.8 | 1.2 | 96.5 | 0.98 | 0.61 | 0.99 | 0.60 |
| **Health system utilization** | |  |  |  |  |  |  |  |  |  |  |
|  | Hospitalization | 5.7 | 6.7 | 4.2 | 1.5 | 2.5 | 91.8 | 0.96 | 0.67 | 0.98 | 0.65 |
|  | Emergence room visit | 12.0 | 13.8 | 7.9 | 4.1 | 5.9 | 82.1 | 0.90 | 0.61 | 0.94 | 0.56 |
|  | Dentistry services | 38.8 | 36.4 | 28.5 | 10.2 | 7.9 | 53.3 | 0.82 | 0.76 | 0.85 | 0.61 |
|  | Health examination | 4.4 | 5.5 | 1.9 | 2.5 | 3.5 | 92.0 | 0.94 | 0.39 | 0.97 | 0.36 |
|  | Overall | 15.2 | 15.6 | 10.6 | 4.6 | 5.0 | 79.8 | 0.90 | 0.69 | 0.94 | 0.63 |
