# Supplementary material for: Concordance between Patient Self-Reports and Claims Data on Clinical Diagnoses, Medication Use, and Health System Utilization in Taiwan
Source: PLoS One. 2014 Dec 2;9(12):e112257. doi: 10.1371/journal.pone.0112257 (PMC4251897; doi:10.1371/journal.pone.0112257)
Supplement: Table S4 — Concordance between self-report and claims record, by diagnoses, medication use, and health system utilization among participants aged 50 years and older. (DOC) [file pone.0112257.s004.doc]

Supplemental Table S4. Concordance between self-report and claims record, by diagnoses, medication use, and health system utilization among participants aged 50 years and older

|  |  | Self-reports (%) | Claims records (%) | In claims records, in self-reports (%) | In self-reports only (%) | In claims records only (%) | Not in claims records, not in self-reports (%) | Total agreement | Positive agreement | Negative agreement | Kappa |
| --- | --- | --- | --- | --- | --- | --- | --- | --- | --- | --- | --- |
| **Diagnoses** | |  |  |  |  |  |  |  |  |  |  |
|  | Hypertension | 31.5 | 36.3 | 25.7 | 5.7 | 10.5 | 58.0 | 0.84 | 0.76 | 0.88 | 0.64 |
|  | Diabetes | 13.4 | 16.2 | 11.8 | 1.6 | 4.4 | 82.2 | 0.94 | 0.80 | 0.96 | 0.76 |
|  | Dyslipidemia | 22.4 | 16.7 | 8.7 | 13.7 | 8.0 | 69.6 | 0.78 | 0.45 | 0.87 | 0.31 |
|  | Malignancy | 1.9 | 4.0 | 1.7 | 0.2 | 2.3 | 95.8 | 0.97 | 0.58 | 0.99 | 0.56 |
|  | Stroke | 3.9 | 7.9 | 2.7 | 1.3 | 5.2 | 90.8 | 0.93 | 0.45 | 0.97 | 0.42 |
|  | Asthma | 3.6 | 5.2 | 1.8 | 1.8 | 3.4 | 93.0 | 0.95 | 0.41 | 0.97 | 0.39 |
|  | Chronic pulmonary diseases | 4.6 | 11.6 | 2.2 | 2.3 | 9.4 | 86.0 | 0.88 | 0.28 | 0.94 | 0.22 |
|  | Gout | 6.7 | 7.9 | 3.3 | 3.4 | 4.6 | 88.7 | 0.92 | 0.45 | 0.96 | 0.41 |
|  | Osteoporosis | 11.1 | 6.1 | 2.7 | 8.4 | 3.4 | 85.5 | 0.88 | 0.31 | 0.94 | 0.25 |
|  | Arthritis | 8.9 | 18.4 | 4.7 | 4.2 | 13.7 | 77.4 | 0.82 | 0.35 | 0.90 | 0.26 |
|  | Renal diseases | 7.0 | 8.0 | 3.0 | 4.0 | 5.0 | 88.0 | 0.91 | 0.40 | 0.95 | 0.35 |
|  | Heart diseases | 10.4 | 23.1 | 7.7 | 2.6 | 15.4 | 74.3 | 0.82 | 0.46 | 0.89 | 0.37 |
|  | Chronic hepatitis | 4.7 | 11.1 | 3.1 | 1.6 | 7.9 | 87.4 | 0.91 | 0.40 | 0.95 | 0.36 |
|  | Psychiatric disorders | 2.0 | 12.3 | 1.1 | 0.8 | 11.2 | 86.8 | 0.88 | 0.16 | 0.94 | 0.13 |
|  | Overall | 9.4 | 13.2 | 5.7 | 3.7 | 7.5 | 83.1 | 0.89 | 0.51 | 0.94 | 0.45 |
| **Medication use** | |  |  |  |  |  |  |  |  |  |  |
|  | Anti-hypertensives | 28.0 | 32.5 | 23.0 | 5.0 | 9.5 | 62.5 | 0.85 | 0.76 | 0.90 | 0.66 |
|  | Anti-diabetes | 12.1 | 12.8 | 10.6 | 1.5 | 2.2 | 85.8 | 0.96 | 0.85 | 0.98 | 0.83 |
|  | Lipid lowering agents | 9.6 | 8.7 | 4.1 | 5.5 | 4.6 | 85.8 | 0.90 | 0.45 | 0.94 | 0.39 |
|  | Anti-asthmatics | 3.0 | 4.5 | 1.5 | 1.5 | 3.0 | 94.0 | 0.95 | 0.40 | 0.98 | 0.38 |
|  | Anti-gout drugs | 6.1 | 5.5 | 2.4 | 3.6 | 3.1 | 90.8 | 0.93 | 0.42 | 0.96 | 0.38 |
|  | Overall | 11.8 | 12.8 | 8.3 | 3.4 | 4.5 | 83.8 | 0.92 | 0.68 | 0.95 | 0.63 |
| **Health system utilization** | |  |  |  |  |  |  |  |  |  |  |
|  | Hospitalization | 12.8 | 12.9 | 9.9 | 2.8 | 3.0 | 84.3 | 0.94 | 0.77 | 0.97 | 0.74 |
|  | Emergence room visit | 15.9 | 18.2 | 10.4 | 5.5 | 7.8 | 76.4 | 0.87 | 0.61 | 0.92 | 0.53 |
|  | Dentistry services | 36.1 | 35.8 | 26.6 | 9.6 | 9.2 | 54.6 | 0.81 | 0.74 | 0.85 | 0.59 |
|  | Health examination | 17.4 | 20.9 | 8.7 | 8.8 | 12.3 | 70.3 | 0.79 | 0.45 | 0.87 | 0.32 |
|  | Overall | 20.5 | 22.0 | 13.9 | 6.7 | 8.1 | 71.4 | 0.85 | 0.65 | 0.91 | 0.56 |
