# Supplementary material for: Concordance between Patient Self-Reports and Claims Data on Clinical Diagnoses, Medication Use, and Health System Utilization in Taiwan
Source: PLoS One. 2014 Dec 2;9(12):e112257. doi: 10.1371/journal.pone.0112257 (PMC4251897; doi:10.1371/journal.pone.0112257)
Supplement: Table S5 — Factors associated with disagreement between self-report and claims record in hypertension, diabetes mellitus, dyslipidemia, and psychiatric disorders. (DOC) [file pone.0112257.s005.doc]

Supplemental Table S5. Factors associated with disagreement between self-report and claims record in hypertension, diabetes mellitus, dyslipidemia, and psychiatric disorders

|  |  | Hypertension | |  | Diabetes |  |  | Dyslipidemia | |  | Psychiatric disorders | | |  |  |  |  |  |  |
| --- | --- | --- | --- | --- | --- | --- | --- | --- | --- | --- | --- | --- | --- | --- | --- | --- | --- | --- | --- |
|  |  | Odds ratio | (95% CI) | | Odds ratio | (95% CI) | | Odds ratio | (95% CI) | | Odds ratio | (95% CI) | |  |  |  |  |  |  |
| Age group (vs. 12-29) | |  |  |  |  |  |  |  |  |  |  |  |  |  |  |  |  |  |  |
| 30-49 |  | 4.43 | (3.07 | , 6.40) | 3.43 | (1.82 | , 6.47) | 2.80 | (2.21 | , 3.56) | 2.68 | (2.01 | , 3.56) |  |  |  |  |  |  |
| ≥50 |  | 15.13 | (10.28 | , 22.27) | 10.62 | (5.50 | , 20.50) | 5.63 | (4.35 | , 7.28) | 4.77 | (3.48 | , 6.54) |  |  |  |  |  |  |
| Gender |  |  |  |  |  |  |  |  |  |  |  |  |  |  |  |  |  |  |  |
| Male vs. female | | 1.27 | (1.11 | , 1.46) | 1.11 | (0.90 | , 1.39) | 1.08 | (0.97 | , 1.21) | 0.65 | (0.57 | , 0.75) |  |  |  |  |  |  |
| Education (vs. ≤6 years)^a^ | |  |  |  |  |  |  |  |  |  |  |  |  |  |  |  |  |  |  |
| 7-12 |  | 0.64 | (0.53 | , 0.76) | 0.70 | (0.53 | , 0.91) | 0.81 | (0.70 | , 0.93) | 0.79 | (0.66 | , 0.95) |  |  |  |  |  |  |
| ≥13 |  | 0.65 | (0.52 | , 0.81) | 0.60 | (0.42 | , 0.86) | 1.13 | (0.96 | , 1.34) | 0.66 | (0.53 | , 0.82) |  |  |  |  |  |  |
| Marriage^a^ (vs. single) | |  |  |  |  |  |  |  |  |  |  |  |  |  |  |  |  |  |  |
| Married/living as married | | 1.00 | (0.75 | , 1.32) | 1.39 | (0.85 | , 2.29) | 1.67 | (1.35 | , 2.06) | 1.07 | (0.83 | , 1.38) |  |  |  |  |  |  |
| Divorced/widowed/separated | | 0.84 | (0.58 | , 1.21) | 1.15 | (0.62 | , 2.14) | 1.40 | (1.05 | , 1.86) | 1.16 | (0.83 | , 1.63) |  |  |  |  |  |  |
| Residence (vs. urban) | |  |  |  |  |  |  |  |  |  |  |  |  |  |  |  |  |  |  |
| Sub-urban | | 1.13 | (0.97 | , 1.31) | 1.00 | (0.79 | , 1.27) | 0.89 | (0.80 | , 1.00) | 0.92 | (0.79 | , 1.06) |  |  |  |  |  |  |
| Rural |  | 1.14 | (0.94 | , 1.38) | 1.16 | (0.87 | , 1.55) | 0.91 | (0.78 | , 1.06) | 1.01 | (0.84 | , 1.22) |  |  |  |  |  |  |
| Body mass index (vs. <24) | |  |  |  |  |  |  |  |  |  |  |  |  |  |  |  |  |  |  |
| Overweight (BMI 24-26 | | 0.99 | (0.84 | , 1.18) | 1.34 | (1.02 | , 1.76) | 1.79 | (1.57 | , 2.04) | 0.99 | (0.84 | , 1.16) |  |  |  |  |  |  |
| Obesity (BMI ≥ 27) | | 1.63 | (1.40 | , 1.91) | 2.25 | (1.76 | , 2.88) | 2.58 | (2.28 | , 2.93) | 0.95 | (0.80 | , 1.13) |  |  |  |  |  |  |

Note:

^a^ Significance was determined using the multivariate generalized estimating equation by Wald Z test with df = 1 and was declared as *p*<0.05.
